# Quantum Interference and Selectivity through Biological Ion Channels

**DOI:** 10.1038/srep41625

**Published:** 2017-01-30

**Authors:** Vahid Salari, Hamidreza Naeij, Afshin Shafiee

**Affiliations:** 1 Department of Physics, Isfahan University of Technology, Isfahan 84156-83111, Iran; 2 School of Physics, Institute for Research in Fundamental Sciences (IPM), P.O. Box 19395-5531, Tehran, Iran; 3 Research Group on Foundations of Quantum Theory and Information, Department of Chemistry, Sharif University of Technology, P.O. Box 11365-9516, Tehran, Iran

## Abstract

The mechanism of selectivity in ion channels is still an open question in biology for more than half a century. Here, we suggest that quantum interference can be a solution to explain the selectivity mechanism in ion channels since interference happens between similar ions through the same size of ion channels. In this paper, we simulate two neighboring ion channels on a cell membrane with the famous double-slit experiment in physics to investigate whether there is any possibility of matter-wave interference of ions via movement through ion channels. Our obtained decoherence timescales indicate that the quantum states of ions can only survive for short times, i.e. ≈100 picoseconds in each channel and ≈17–53 picoseconds outside the channels, giving the result that the quantum interference of ions seems unlikely due to environmental decoherence. However, we discuss our results and raise few points, which increase the possibility of interference.

Recently, the quantum world has opened up new perspectives in the field of complex systems and biology^1^,^2^,^3^,^4^,^5^,^6^. Energy, charge, or information transfer are important phenomena in physical and biological systems taking place at scales ranging from atoms to large macro-molecular structures, and the idea has been put forward that quantum mechanics might have a positive effect on the efficiency of energy or charge transport in such systems. Energy transfer in photosynthetic structures^7^,^8^,^9^,^10^, avian quantum compass in migratory birds^11^, and charge transport through DNA^12^ are good examples in this context. The living cell is an information replicating and processing system that is replete with naturally-evolved nanomachines, which at some level may require a quantum mechanical description despite critical limitations for application of quantum theory in biology^13^. In this case, ion channels are good examples in living cells in which quantum effects may play a role^14^,^15^,^16^,^17^,^18^,^19^,^20^. They are proteins in the membrane of cells that can cooperate for the onset and propagation of electrical signals across membranes by providing a highly selective conduction of charges bound to ions through a channel like structure. In fact, each ion channel is specialized for specific ions, e.g. potassium channels only permit potassium ions to pass the membrane while they reject other ions (e.g. sodium) to pass. This property is called selectivity and the important part of the ion channel, which is responsible for selectivity, is called selectivity filter. Numerous investigations of ion selectivity have been conducted over more than 50 years, yet the mechanisms whereby the channels select certain ions and reject others are not well understood^21^. The selectivity filter is a part of the protein forming a narrow tunnel inside the ion channel which is responsible for the selection process and fast conduction of ions across the membrane^22^. The 3.4 nanometer long KcsA channel (i.e. potassium crystallographically-sited activation channel) is comprised of a 1.2 nanometer long selectivity filter that is composed of four P-loop strands. The Carbonyl groups (i.e. C=O’s) are responsible for trapping and displacement of ions in the filter (see Fig. 1).

Here, we would like to investigate the possibility of matter-wave interference of ions via passing the ion channels. This possibility can be important as it may explain the “selectivity” property in ion channels because interference occurs between similar particles through similar slits with specific distances between them.

Our paper is organized as follows: we first introduce our simulation for two neighboring ion channels as a double-slit and then briefly investigate the possibility of matter-wave interference through the slits. Then, we estimate the plausible distances between the ion channels to produce ionic interference. In the next step, we consider the effect of environmental decoherence on quantum states of ions inside and outside the slits, and accordingly we obtain the coherence lengths of ions for making interference. Finally, we summarize and discuss our results.

## A Simulation for two neighboring ion channels as a double-slit

Feynman believed that we can see the whole mysteries of quantum theory in the double-slit experiment^23^. Basically, quantum physics is centered on microscopic phenomena with photons, electrons and atoms, however objects of increasing complexity have attracted a growing scientific interest recently. For example, the matter-wave interference has been investigated theoretically and experimentally confirmed for large molecules such as C_60_ (i.e. 60 atoms), Tetraphenylporphyrin (i.e. 78 atoms) and functionalized Tetraphenylporphyrin (i.e. 810 atoms) in double-slit and gratings interferometers^24^,^25^,^26^,^27^,^28^,^29^. Now the question is: are these effects applicable in biology, too? If yes, can such matter-wave effects play any role in biological functions?

In this section, we would like to apply a simulation for quantum interference through neural ion channels. We expect to see quantum effects appearing due to small dimensions of the selectivity filter during the crossing of ions through ion channels. One of the quantum effects is the matter-wave interference of ions, which may show some quantum roots for action potential production in excitable cells (e.g. neurons).

## Speed and wavelength of ions inside the selectivity filter

Here, we would like to obtain the velocity and consequently the de Broglie wavelength of ions (i.e. *λ*_dB_ = h/*mv* where h is the Planck’s cnstant, *m* is the mass of ion, and *v* is the velocity of ion) inside the selectivity filter via molecular dynamics (MD) simulation. In fact, our method (see Supplementary Information) is the same as what we used previously^17^,^20^ but we use it here with higher cutoffs, i.e. 1 nm for the van der Waals interaction and 1 nm for electrostatic interactions. Taking typical values for membrane potentials in neurons, −70 mV and +30 mV, for resting and firing states are considered^30^,^31^. To be more general, we also considered other membrane potentials −100 mV and +100 mV 32 to obtain other velocities as well^17^,^20^. The obtained results are shown in the Table 1, in which the order of the wavelength of ions (*λ*_0_ ≈ 0.1 nm) is the same as the order of the width of each slit (i.e. 0.3 nm), *O(λ*_0_) ≈ *O(b*), which is the necessary condition for interference.

## The distance between ion channels

**Figure 2:**
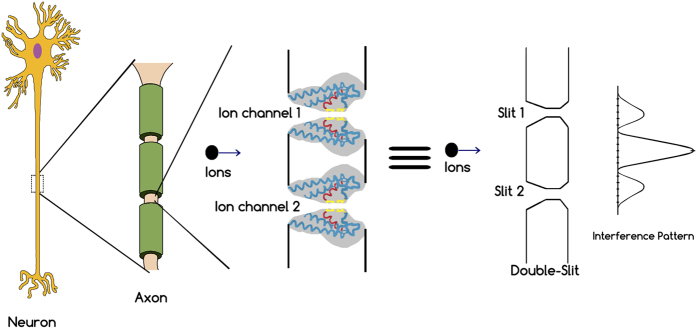
(Left) Ions passing through two neighboring ion channels on a cell membrane in a neuron, (Right) Simulation of two neighboring ion channels as a double-slit interferometer.

Now, we simulate two neighboring ion channels on a neural cell membrane with a double-slit experiment (see Fig. 2).

Here, we consider potassium ions passing the KcsA ion channels by focusing on the selectivity filter structure. As a matter of fact, ions act as wavepackets in the filter since they are trapped by carbonyl groups in the filter and consequently getting longer de Broglie wavelengths according to our MD simulation. We use the “macroscopicity” method^33^ to obtain the possible distances between the slits because in this method it is supposed that ion behaves like a Gaussian state, and after the slits the ion behaves like a free particle in two directions *x* and *y*. To clarify the macroscopicity method, we first introduce the dimensionless form of the Schrodinger equation. This method is described in more details in ref. 33 (see Supplementary Information) for obtaining interference pattern. In the dimensionless regime, we introduce the characteristic parameters for length R_0_ and energy U_0_ as constant units of length and energy of a quantum system, respectively. Also, for an ion mass, *M*, one can introduce the characteristic time as *τ*_0_ = R_0_/(U_0_/*M*)^1/2^. Since, U_0_ acts like the kinetic energy of quantum system, the unit of momentum could be expressed as 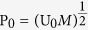. Subsequently, the conjugate variables of position *q* and momentum *p* are defined as *q* = *R*/R_0_ and *p* = *P*/P_0_ where *R* and *P* are the conventional position and momentum, respectively^34^.

Moreover, the potential energy 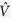 and the Hamiltonian 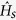 operators of the system can be defined in this regime as


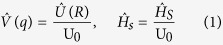


where 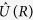 and 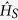 are the potential energy and the Hamiltonian operators in the ordinary Schrodinger equation. Finally, the dimensionless Schrodinger equation can be written as


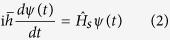


Also, the canonical commutator in the dimensionless form is 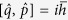, where 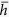 defined as


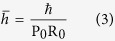


**Figure 3:**
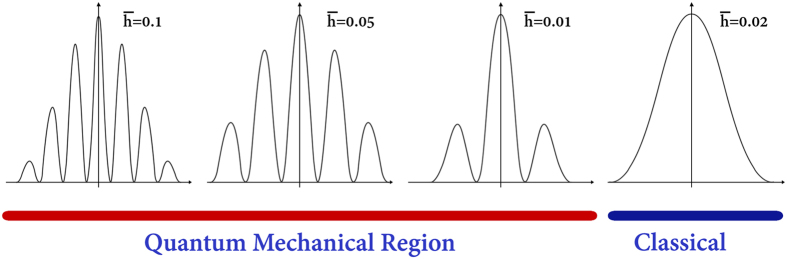
The macroscopicity measure, 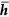, indicates the region of quantum coherence for interference. The values lower than 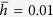 are classical values that show the interference pattern cannot be formed. The values from 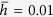 to 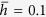 are quantum values which makes the interference pattern possible.

So far, many studies have been done to investigate double-slit interference pattern of particles, atoms and molecules in experimental and theoretical contexts. In some of these works, the incoming state in double-slit experiment has been described by Gaussian wave packets^35^,^36^. Our approach in this study is based on Gaussian wave packet as a simulation for potassium ions which move through two neighboring ion channels in two dimensions. The use of Gaussian wave packet is sufficiently general, because it includes the limit case of plane waves. On the other hand, due to the development of experimental techniques, possible deviations from the standard form of the interference pattern can be better explained by Gaussian states^37^,^38^,^39^. As we mentioned above, the macroscopicity method is used for obtaining interference patterns by using a dimensionless form of the Schrodinger equation in which a new dimensionless parameter 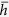 appears showing quantitatively the quantum behavior of the system. We can define 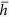 as (see Supplementary Information)


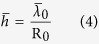


where 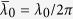. Here, *λ*_0_ is the de Broglie wavelength of the system. Strictly speaking, the situation in which one obtains 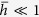, the system behaves quasi-classically. The values of 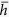 between 0.01 to 0.1 are fair enough to show the macroscopic disposition of the proposed system to behave quantum mechanically^34^. If we let *λ* ≈ 0.1 nm as the average de Broglie wavelength of the ion inside the selectivity filter and then let *R*_0_ ≈ 0.2 nm as the size (or diameter) of the ion then 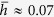 which falls in the quantum region. Now, we obtain the real values of the distance between two neighboring ion channels, *d*′, according to different measures of 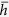. For this purpose, we draw the interference patterns according to the equation Supplementary Information - 13 in which *b* is the width of selectivity filter, and *d* is a variable parameter similar to the real range of distances between ion channels for potassium ion. Notice that we use dimensiomless form of *b* and *d* for drawing of the interference patterns. In the dimensionless regime, *d* can be defined as *d* = *d*′/R_0_ and *b* = *b*′/R_0_ where *d*′ is the distance between the slits, *b*′ is the width of selectivity filter (i.e. 0.3 nm) and R_0_ is the size of potassium ion (≅0.2 nm) as a characteristic parameters for length. For interference patterns in the regions of our investigation, Figures (SI-1–SI-4) and Fig. 3, the different values of the distance between ion channels in relation with macroscopicity measure 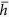 are given in Table 2. Based on our results, the macroscopicity measure, 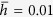 is approximately a threshold for the distances between the ion channels for interference. It means the maximum double-slit distance between the slits is roughly *d*′ = 5.90 nm. The other *d*′ values obtained from quantum values 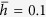 (*d*′ = 0.18 nm) and 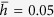 (*d*′ = 0.38 nm) are not biologically feasible.

In the following section we will investigate the effect of biological environment on the quantum states of ions inside and outside of the selectivity filter. Then, we will obtain the coherence length of ions outside the selectivity filter.

## Decoherence

The biological system is a very noisy and hot environment for quantum states and therefore there is a serious problem against quantum interference. In fact, “high vacuum is a prerequisite for all matter-wave interferometers”^29^. Additionally, in most interferometers vibrations are important source of dephasing^29^. Based on quantum mechanics and the decoherence model, every system rapidly entangle with the surrounding environment, which causes dephasing of their quantum states. Now, the question is: how ions can keep their quantum states during the whole crossing mechanism through ion channels while they face environmental particles in the hot, wet and noisy environment of the cell? Normally, crossing of each ion through ion channels takes at least 10–20 ns^40^ and therefore the decoherence time should be at least bigger than this time interval. This is rather a big decoherence time from quantum mechanical point of view and consequently it is a fundamental problem against the matter-wave interference of ions in biological environment. Considering the fact that even delocalized electrons (with much lighter mass than potassium ions) in low temperature quantum dots will dephase in ≈1 ns, how would it be feasible for massive potassium ions to remain coherent, showing interference patterns over 10–20 ns time scale? Tegmark already did very interesting calculations about decoherence timescales in neurons^30^ and obtained that the decoherence times are very short (≈10^−20^ s) for superposed ions to be effective in any cognitive processes. In fact, Tegmark didn’t consider the ion channel structure at all and his model was very simple as he only considered the superposition distance of ions equal to the membrane thickness. We already corrected the Tegmark’s calculations^30^ and obtained the decoherence timescales for a single ionic superposition in a single selectivity filter, which was in order of picoseconds, i.e. a hundered million times bigger than what he obtained^17^. Additionally, the Tegmark’s decoherence rate function leads to decoherence times that are directly proportional to temperature indicating his calculations do not address the temperature dependence of decoherence times correctly^41^,^42^. Despite the general controversial aspects of the decoherence model^41^,^43^,^44^ we would like to use the standard approach to investigate the decoherence timescales to see how fast an ionic superposition becomes decohered as a consequence of vibrations and environmental scattering.

## Decoherence Formulation

Scattering with environmental particles is the most important reason of decoherence in every quantum system. The time evolution of a quantum superposed state can be shown by a reduced density matrix *ρ(x, x*′, *t*) in the following form


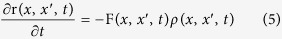


where the *ρ(x, x*′, *t*) is the density matrix of system in terms of time and position at time *t, ρ(x, x*^′^, 0) is the density matrix at *t* = 0, and F(*x, x*′, *t*) is the decoherence factor as follows^45^


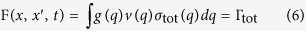


where *g(q*) is the number density of scatterers with momentum *q* that ∫*g(q)dq* = *N/V*, in which *N* is the number of scatterers and *V* is the volume, *v(q*) = *q/m*′ is the speed of scatterers with mass *m*′ and momentum *q, σ*_tot_(*q*) is the scattering cross section for the momentum *q*, and Γ_tot_ is the total scattering rate. Thus, we can rewrite the equation (5) in the following form


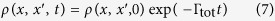


In general, the evolution of a quantum system regarding the decoherence can be written in the form of


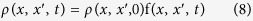


where f(*x, x*′, *t*) is the delocalization rate of the superposed state in terms of time and position^45^. For scattering of environment particles that have a typical de Broigle wavelength *λ*_en_, we have^30^,^46^


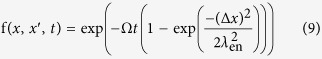


where Ω = *σϕ* in which *σ* is the scattering cross section and *ϕ* = *nv* is the flux, in which *n* is the density of scatterers and *v* is the velocity of object^17^, and Δ*x* = |*x* − *x*′| is the superposition distance of the object. In the short wavelength limit, Δ*x*  ≫  *λ*_en_, we can approximately rewrite^30^


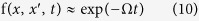


On the other side, we have a time-dependent relation for density matrix 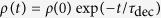
45. Thus, we have *τ*_dec_ = 1/Ω and as *λ*_0_ = h/*mv* (i.e. de Broglie’s relation for ion) we can finally write


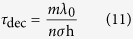


where here *m* is the mass of the ion, *λ*_0_ is the wavelength of the ion, *n* is the density of scatterers (*n* = *N/V* with *N* the number of scatterers and *V* is the volume).

In the long wavelength limit, *λ*_en_  ≫ Δ*x*, the decoherence time in this case can be obtained from the equation (9) giving^17^


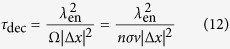


It is obvious that we are in the short wavelength limit because the superposition distance Δ*x* = 5.9 is much bigger than the environmental de Broglie wavelength of particles, which are in the range 0.02–0.05 nm. Thus, we should calculate the decoherence times inside and outside the selectivity filter according to the equation 11. In this case, the decoherence time does not depend on the superposition distance.

## Decoherence inside the selectivity filter

**Figure 4:**
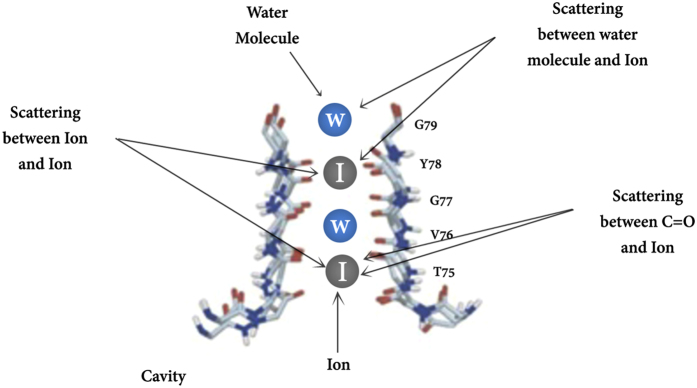
The quantum states of ions in the selectivity filter may be decohered due to scattering with environmental particles and biomolecules in biological temperature.

Assume that the selectivity filter is a cavity with volume *V* including *N* particles. The ion is the system which can be scattered by the particles in the cavity. The main scattering can happen between the ion and the particles in the cavity such as C=O bounds, water molecules and other ions (see Fig. 4). The decoherence time is obtained by letting *n* = *N/V* into the equation 11 as follows


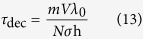


The volume of the cavity as a cylinder is *V* = *πr*^2^*L* where *r* is the radius of the cavity and *L* is the length of the cavity. We already calculated the decoherence times in the filter^17^ based on the volume of the selectivity filter with radius *r* = 0.15 nm, however in this volume the C=O bonds cannot play the role as scatterers since they are behind the above radius. In fact, the whole radius in which carbonyl groups can be considered as scatterers in a cavity is approximately *r* = 0.4 nm. The scattering cross section can be approximately obtained from *σ* ≈ *πa*^2^ where *a* is the radius of the ion.

The results are shown in the Table 3. The results indicate that the decoherence time is around 100 picoseconds (i.e. ≈0.1 ns) inside the selectivity filter mainly due to scattering with C=O bonds. However, we should also consider that the selectivity filter backbone has vibrations that means the above cavity is vibrating. As we mentioned before, one of the important sources of dephasing can be due to channels vibrations in biological temperature. In fact, if ions are delocalized in the two channels then the vibrations of each channel can give random noise to the wave function and decohere it. We should notice here that the membrane fluctuations vary between 0.3–30 Hz^47^ and consequently the periods of vibrations vary between 0.03–3.3 sec which are much bigger than the pico– nano seconds timescales of the ionic translocation times. Therefore, at this scale the vibrations are not so effective. Indeed, the ion channel creates a fluctuating potential and coupling to phonons will be likely to dephase the quantumness in the system. If a particular system–environment interaction leads to dissipation in the system, then the strength of the system–environment interaction is a measure of the relaxation time. As the interaction strength decreases, the relaxation times become longer, and vice versa. The relation between the relaxation time and decoherence time for an object is^45^


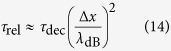


where *λ*_dB_ is the thermal de Broglie wavelength of the ion. Based on our MD simulation, we let *λ*_dB_ ≈ 0.1 nm. The estimations for the relaxation times are shown in Table 3. It is seen that the relaxation times are mainly in order of nanoseconds but the decoherence times are in order of picoseconds. The obtained decoherence time (i.e. 0.1 ns) is a hundred times shorter than the ion translocation time in the filter (i.e. 10–20 ns), which makes the delocalization of ions in the two channels unlikely.

In the following subsection, we will obtain the decoherence times outside the channels.

## Decoherence outside the selectivity filter

Basically each ion is bounded by eight water molecules outside the selectivity filter^48^. Considering thermal energy at room temperature, k_B_*T* = 4.11 × 10^−21^ J, the velocity of the hydrated water will be 
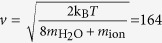
 m/s, thus the de Broglie wavelength of the ion is 
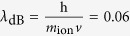
 nm. The speed of potassium ion (in biological temperature) that is not hydrated is 
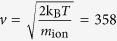
 m/s. Thus, its de Broglie wavelength is *λ*_dB_ = 0.02 nm.

The maximum superposition distance should be Δ*x* = 5.9 nm for the maximum distance between the ion channels. Thus, for the superposition state of the ion outside of the selectivity filter and regarding the delocalization of the ion between the two ion channels we should consider the Eq. 11, which is used in the short wavelength limit (i.e. Δ*x*  ≫ *λ*_en_) for scattering between the system and the environmental particles. For the ion density, let us write 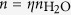 where the density of water molecules 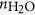 is about (1 g/cm^3^)/(18 *m*_*p*_) ≈ 10^23^/cm^3^ = 10^27^/m^3^ and *η* is the relative concentration of ions (positive and negative combined)^30^. Typical ion concentrations during the resting state are [*Na*^+^] = 9.2 mmol/l = 92 × 10^16^/cm^3^ for inside and [*Na*^+^] = 120 mmol/l = 12 × 10^18^/cm^3^ outside the axon membrane and [*K*^+^] = 140 mmol/l = 14 × 10^18^/cm^3^ for inside and [*K*^+^] = 2.5 mmol/l = 25 × 10^16^/cm^3^ outside the axon membrane^30^.

Letting *σ* ≈ 10^−20^ m^2^ we obtain the decoherence times as in the Table 4. The results indicate that the superposition states can only survive about 17–53 picoseconds.

## Discussion

In previous sections, we have obtained decoherence times of ions inside and outside the selectivity filter. The decoherence time inside the filter is about 100 ps, which is at least a hundred times shorter than 10–20 ns translocation time in the filter, making interference unlikely. However, the average velocity of ion in the filter is *v* ≈ 100 m/s and the length of selectivity filter is *L* ≈ 1.2 nm. We expect the translocation time to be *t* = *L/v* ≈ 10 ps but in reality the translocation time is 1000 times bigger than this time. It means that ions are trapped in the filter. We have already shown that there is a substantial “cooling effect” of ions in the filter by solving Schrodinger equation for ions and the electric potential in the selectivity filter^19^, indicating that ions are strongly trapped by carbonyl groups via electric interactions. This can make a weaker scattering with C=O bonds and therefore longer decoherence times. In fact, the present obtained decoherence times in this paper are based on the classical MD simulation that ignores the quantum effects. In order to have a better analysis, the ion movement in the filter should be investigated by quantum simulations or QM/MM (quantum mechanics/molecular mechanics) methods^49^.

Regarding the ps decoherence timescales outside the filter, we should calculate the coherence length as well. Based on the data for both isolated and hydrated ions and their velocities outside the selectivity filter as well as their decoherence times (see Table 4) we calculated the coherence length of ions,


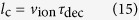


where *l*_c_ is the coherence length of ion, and *v*_ion_ is the velocity of ion.

The results indicate that the coherence length varies between 6.2–8.5 nm that is biologically feasible for interference but very close to the membrane. However, we should also compare the obtained coherence length with the mean free path (MFP) of ions regarding their collisions with water molecules around. The MFP is obtained as follows^50^


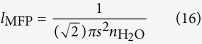


where *πs*^2^ is the effective collision area, and *s* is the size of water molecule (≈0.25 nm), which gives *l*_MFP_ ≈ 5.1 nm. The obtained coherence lenghts and MFP are seen in the Table 5 in which the coherence lengths are close to the MFP, indicating that only one collision may change the direction of a superposed ion during the displacement of ions to the channels. This result makes interference plausible outside the filter despite short decoherence timescales.

## Conclusion

In this paper, we investigated the possibility of quantum interference of ions through ion channels to see whether quantum interference can be the cause of selectivity in ion channels. Regarding the selectivity property, the main question is: what properties make the ion channels so selective? A convincing theory has to explain how a channel can permit passage of a particular ion, while excluding all ions of smaller diameter, including some that are much smaller^51^. To answer this question we suggested that the matter-wave interference can be a solution for selectivity, since interference happens between similar ions regarding the same size of slits. Additionally, quantum interference can make the transport of ions faster (i.e. 10^8^ ions per second) as the ions don’t choose wrong channels. Here, we have investigated potassium ions passing the two neighboring KcsA ion channels via simulation with the physical double-slit experiment. Our results can be summerized as follows: (1) There is an estimated upper bound of 5.9 nm for inter-channel distance in order potentially to obtain quantum interference, (2) quantum states can cohere for about 100 picoseconds inside the selectivity filter, (3) coherence can be maintained for about 17–53 picoseconds outside the filter, and (4) the coherence length of an ion varies between 6.2–8.5 nm outside the filter, which are close distances to the membrane. Despite the feasibility of coherence length outside the filter, our present estimations inside the filter indicate that quantum interference seems unlikely. We have discussed above that ions can be trapped and cooled in the filter based on our previous quantum simulation^19^, thus the decoherence time can be increased due to weaker scattering effects.

Testing our hypothesis seems a difficult task in real conditions of the cell, but we would like to motivate other research teams to evaluate our approach experimentally. For example, it may be possible with the aid of mass spectroscopy techniques to see if any pattern can be formed. Also, it is useful to design a double slit experiment in the lab and simulate the similar conditions of the cell with the same size of channels in nano dimensions and investigate the interference patterns by counting ions at different places after the double-slit.

## Additional Information

**How to cite this article**: Salari, V. *et al*. Quantum Interference and Selectivity through Biological Ion Channels. *Sci. Rep.* **7**, 41625; doi: 10.1038/srep41625 (2017).

**Publisher's note:** Springer Nature remains neutral with regard to jurisdictional claims in published maps and institutional affiliations.

## Supplementary Material

Supplementary Information

## Figures and Tables

**Figure 1:**
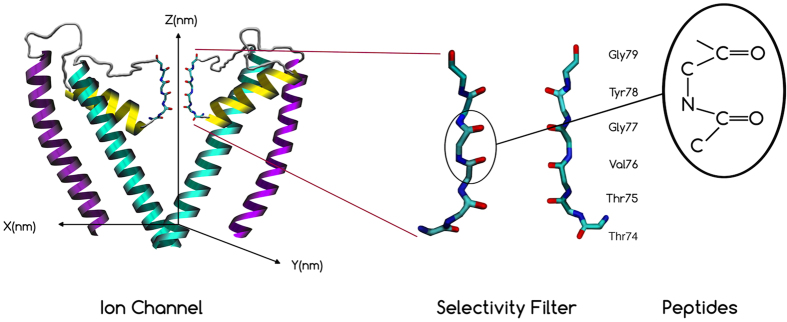
(Left) A representation of KcsA ion channel. (Right) Two P-loop monomers in the selectivity filter, composed of the sequences of TVGYG amino acids [T(Threonine, Thr75), V(Valine, Val76), G(Glycine, Gly77), Y(Tyrosine, Tyr78), G(Glycine, Gly79)] linked by peptide units H-N-C =O^20^.

**Table 1:** The obtained average velocities and wavelenghts of potassium ions via MD simulation.

Ion	Average velocity (m/s), MD simulation			
	*V* = 30 mV	*V* = 100 mV	*V* = −70 mV	*V* = −100 mV
K	100.89	101.61	99.91	98.94
**Ion**	**Wavelenghts of Ion, λ_dB_ = h/*mv* (nm)**			
	*V* = 30 mV	*V* = 100 mV	*V* = −70 mV	*V* = −100 mV
K	0.1120	0.1112	0.1131	0.1142

We have chosen the typical values for membrane potentials in neurons, −70 mV and +30 mV, for resting and firing states as well as −100 mV and +100 mV for a general state^17^,^20^.

**Table 2:** The macroscopicity measure, 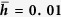 is approximately a threshold for the distances between the ion channels for interference.

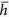 (dimensionless)	*d*′ (nm)
0.1 (Quantum)	0.18
0.05 (Quantum)	0.38
0.01 (Quantum)	5.90
0.002 (Classical)	7.70

It means the maximum double-slit distance between the slits is roughly *d*′ = 5.90 nm.

**Table 3:** The obtained relaxation times and decoherence times for the ion superposition states in the selectivity filter.

System	Environment	N	Δ*x* (nm)	*τ*_rel_ ( 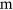 s)	*τ*_dec_ (ns)
K^+^	N*a*^+^	2	0.3	0.009	1.020
K^+^	N*a*^+^	2	5.9	3.550	1.020
K^+^	K^+^	2	0.3	0.009	1.020
K^+^	K^+^	2	5.9	3.550	1.020
K^+^	C=O	20	0.3	0.0009	0.102
K^+^	C=O	20	5.9	0.355	0.102
K^+^	C=O	8	0.3	0.0022	0.127
K^+^	C=O	8	5.9	0.8841	0.127
K^+^	H_2_O	3	0.3	0.0061	0.340
K^+^	H_2_O	3	5.9	2.376	0.340

Here, the filter considered as a cavity in which a few number of particles are present.

**Table 4:** The decoherence times for ionic superposition states outside the selectivity filter.

System	Environment	*λ*_0_ (nm)	n (1 /m^3^)	*τ*_dec_ (s)
K ^+^ (hydrated)	water	0.06	10^27^	52.4 × 10^−12^
K ^+^ (isolated)	water	0.02	10^27^	17.4 × 10^−12^
K ^+^ (hydrated)	potassium	0.06	25 × 10^22^	20.9 × 10^−8^
K ^+^ (isolated)	potassium	0.02	25 × 10^22^	69.9 × 10^−9^
K ^+^ (hydrated)	sodium	0.06	12 × 10^22^	43.7 × 10^−8^
K ^+^ (isolated)	sodium	0.02	12 × 10^22^	14.5 × 10^−8^

The results indicate that the decoherence timescale is approximately 17 ps for isolated water and 52 ps for hydrated water, mainly due to scattering with environmental water molecules.

**Table 5:** The coherence lengths of ions outside the selectivity filter.

System	Speed (m/s)	*τ*_dec_ (s)	*l*_MFP_ (nm)	Coherence length (*l*c) (nm)
K^+^(hydrated)	164	52.4 × 10^−12^	5.10	6.229
K^+^(isolated)	358	17.4 × 10^−12^	5.10	8.593
