# Supplementary material for: Quantum Interference and Selectivity through Biological Ion Channels
Source: Sci Rep. 2017 Jan 30;7:41625. doi: 10.1038/srep41625 (PMC5278555; doi:10.1038/srep41625)
Supplement: Supplementary Information [file srep41625-s1.pdf]

SUPPLEMENTARY INFORMATION

Quantum Interference and Selectivity through Biological Ion Channels

Vahid Salari[1,3,*], Hamidreza Naeij[2], Afshin Shafiee[2,3]

[1] **Department of Physics, Isfahan University of Technology, Isfahan 84156-83111, Iran**
[2] **Research Group on Foundations of Quantum Theory and Information, Department of Chemistry, Sharif University of Technology, P.O.Box 11365-9516, Tehran, Iran**
[3] **School of Physics, Institute for Research in Fundamental Sciences (IPM), P.O.Box 19395-5531, Tehran, Iran**

*** vahidsalari@cc.iut.ac.ir**

Macroscopicity Method

To clarify the macroscopicity method, we first introduce the dimensionless form of the Schrodinger equation. This method is described with more details by Naeij and Shafiee [1] for obtaining interference pattern. Dimensionless regime is explained as follows:

First, we introduce the characteristic parameters for length R_0 and energy U_0 as constant units of length and energy of a quantum system, respectively. Also, for an ion mass, M, one can introduce the characteristic time as $\tau_0 = \mathrm{R}_0/(\mathrm{U}_0/M)^{\frac{1}{2}}$. Since, U_0 acts like the kinetic energy of quantum system, the unit of momentum could be expressed as $\mathrm{P}_0 = (\mathrm{U}_0 M)^{\frac{1}{2}}$. Subsequently, the conjugate variables of position q and momentum p are defined as $q = R/\mathrm{R}_0$ and $p = P/\mathrm{P}_0$ where R and P are the conventional position and momentum, respectively [2].

Moreover, the potential energy $\hat{V}$ and the Hamiltonian $\hat{H}_s$ operators of the system can be defined in this regime as

$$\hat{V}(q) = \frac{\hat{U}(R)}{\mathrm{U}_0}, \quad \hat{H}_s = \frac{\hat{H}_S}{\mathrm{U}_0} \tag{1}$$

where $\hat{U}(R)$ and $\hat{H}_S$ are the potential energy and the Hamiltonian operators in the ordinary Schrodinger equation. Finally, the dimensionless Schrodinger equation can be written as

$$\mathrm{i}\bar{h}\frac{d\psi(t)}{dt} = \hat{H}_s\psi(t) \tag{2}$$

Also, the canonical commutator in the dimensionless form is $[\hat{q}, \hat{p}] = i\bar{h}$, where $\bar{h}$ defined as

$$\bar{h} = \frac{\hbar}{\mathrm{P}_0\mathrm{R}_0} \tag{3}$$

As is clear in (2) and (3), a new dimensionless parameter $\bar{h}$ appears which show quantitavely the quantum behavior of the system. Strictly speaking, the situation in which one obtains $\bar{h} \ll 1$, the system behaves quasi-classically. The values of $\bar{h}$ between 0.01 to 0.1 are fair enough to show the macroscopic disposition of the proposed system [2]. Moreover, we can define $\bar{h}$ in (3) as

$$\bar{h} = \frac{\bar{\lambda}_0}{\mathrm{R}_0} \tag{4}$$

where $\bar{\lambda}_0 = \lambda_0/2\pi$. Here, λ_0 is the de Broglie wavelength of the system. For a macroscopic quantum system, λ_0 is too small compared to R_0, which is nearly a fixed value for known models of potential. Thus, $\bar{h} < 0.1$ seems appropriate condition for our future purposes. Smaller values of $\bar{h}$ show more classical behavior of the macro-system.

Moreover, according to the *particle* aspect of a quantum system, we define $\omega_0 = \tau_0^{-1} = \mathrm{P}_0/\mathrm{R}_0 M$. On the other hand, for the *wave* aspect of a quantum system, we introduce another unit of momentum P'_0 concluded from the phase velocity $v'_0 = \omega_0/k_0 (k_0 = 2\pi/\lambda_0)$, so that, $\mathrm{P}'_0 = M\bar{\lambda}_0\omega_0$. Subsequently, regarding the relation (4), we conclude that

$$\bar{h} = \frac{\mathrm{P}'_0}{\mathrm{P}_0} \tag{5}$$

Since for a macroscopic quantum system, the wave aspect is reduced, therefore $P_0' \ll P_0$. This is another reason of how the parameter of $\bar{h}$ can explain the classical and quantum behavior of the macro-system.

Formulation of Double-Slit Interference Pattern of Potassium Ion via Movement through Ion Channels

So far, many studies have been done to investigate double-slit interference pattern of particles, atoms and molecules in experimental and theoretical contexts. In some of these works, the incoming state in double-slit experiment has been described by Gaussian wave packets [3, 4]. The use of Gaussian wave packet is sufficiently general, because it includes the limit case of plane waves. On the other hand, due to the development of experimental techniques, possible deviations from the standard form of the interference pattern can be better explained by Gaussian states [5–7].

Our approach in this study will be based on Gaussian wave packet as a simulation for potassium ions which move through two neighbor ion channels in two dimensions. We suppose that the state of the ion behaves like a Gaussian state and after the slits, the ion can be behaved as a free particle in two directions x and y.

Moreover, we define the region W that is inaccessible to the ion, assumed to be a subset of the (x, y) plane

$$W = \{(x, y) :\mid x \mid< a, y \in (-\infty, -d - b] \cup [-d, d] \cup [d + b, \infty)\} \tag{6}$$

where b is the slits width with depth $2a$ and d is the distance of the slits.

The potential energy constituent of the Hamiltonian operator of the system at $t \geq 0$ is an infinite step potential which does not permit a potassium ion of arbitrary energy to tunnel the region W. We also suppose that the wave function of the incoming ion to slits is factorized in its x and y components. It is assumed that x and y components of the wave function remain separated during and after passing the slits. We consider a Gaussian wave packet coming from the remote x region with probability distribution centered on a point moving with velocity $\bar{h}k_{0x}$ on the x-axis $(y = 0)$ in dimensionless regime as [1]

$$\psi(x, y, t) = \mu(x, t)\phi(y, t) \tag{7}$$

where

$$\mu(x, t) = [\frac{\alpha}{\pi^{\frac{1}{2}}(1 + i\bar{h}\alpha^2 t)}]^{\frac{1}{2}}\{exp[-\frac{\alpha^2}{2}\frac{(x - x_0 - k_{0x}t)^2}{1 + i\bar{h}\alpha^2 t} + \frac{ik_{0x}}{\bar{h}}(x - x_0) - \frac{ik_{0x}^2 t}{2\bar{h}}]\} \tag{8}$$

and

$$\phi(y, t) = [\frac{\beta}{\pi^{\frac{1}{2}}(1 + i\bar{h}\beta^2 t)}]^{\frac{1}{2}}exp[-\frac{\beta^2}{2}\frac{(y - y_0)^2}{1 + i\bar{h}\beta^2 t}] \tag{9}$$

We define the region in which the action of the Gaussian wave packet is investigated as $I = [d, d + b] \cup [-d - b, -d]$. After the slits, the wave function of the ion in the y-direction evolves as

$$\phi_I(y, t) = \frac{1}{2\bar{h}}\frac{\beta^{\frac{1}{2}}}{\pi^{\frac{5}{4}}}\int_{-\infty}^{+\infty} exp[\frac{i}{\bar{h}}(p_y y - \frac{p_y^2 t}{2})]dp_y \int_I exp[-\frac{i}{\bar{h}}p_y\xi - \frac{\beta^2}{2}(\xi - y_0)^2]d\xi \tag{10}$$

where the first integral is the Fourier transform of time evolution of the wave function in the momentum space and the last integral is the wave function of the ion in the momentum space at $t = 0$.

One can obtain the above wave function by integrating over the variable p_y as

$$\phi_I(y, t) = (\frac{\beta}{2\pi^{\frac{3}{2}}i\bar{h}t})^{\frac{1}{2}}exp[y^2\frac{i}{2\bar{h}t} - y_0^2\frac{\beta^2}{2}]\int_I exp[-\xi^2(\frac{\beta^2}{2} - \frac{i}{2\bar{h}t}) + \xi(y_0\beta^2 - \frac{iy}{2\bar{h}t})]d\xi \tag{11}$$

We suppose that the wave packet reaching the slits is very undetermined in the y-position probability distribution

$$\Delta y = \frac{1}{\beta\sqrt{2}} \gg b \tag{12}$$

By setting $\beta^2 \approx 0$ in (11) and neglecting the term $i\xi^2/2\bar{h}t$ against $iy\xi/2\bar{h}t$ for large values of y (knows as far-field approximation) and after some calculations, the double-slit interference pattern of potassium ion can be obtained as

$$\phi_I(y,t)\phi_I^*(y,t) \cong \frac{2b^2\beta}{\pi^{\frac{3}{2}}\bar{h}t} exp[-\beta^2 y_0^2] \frac{sin^2(by/2\bar{h}t)}{(by/2\bar{h}t)^2} cos^2[\frac{y}{\bar{h}t}(d+\frac{b}{2})] \tag{13}$$

The resulting interference patterns for potassium ions via movement through ion channels are drawn in different values of macroscopicity measure $\bar{h}$ in figure (1)-(4).

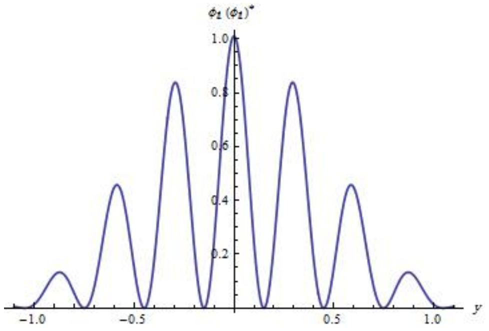

FIG. 1: $\bar{h} = 0.1$

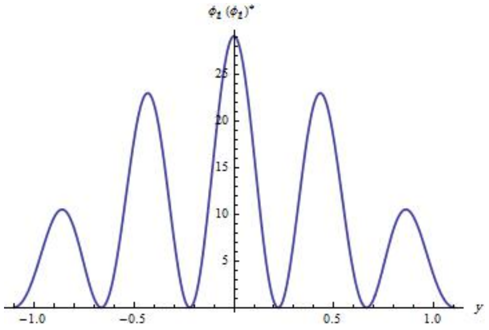

FIG. 2: $\bar{h} = 0.05$

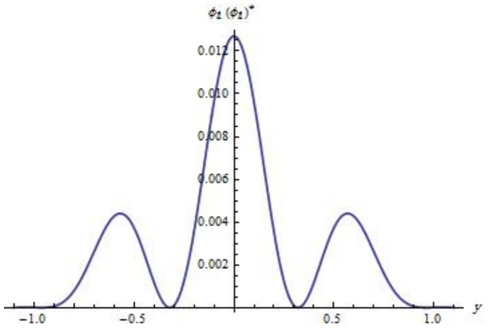

FIG. 3: $\bar{h} = 0.01$

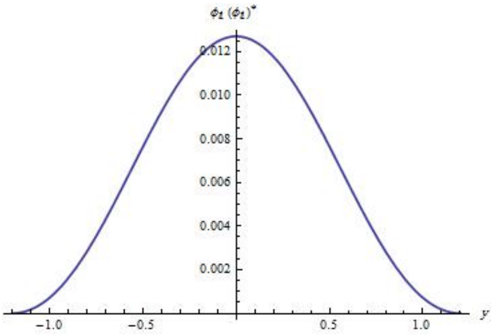

FIG. 4: $\bar{h} = 0.002$

As mentioned before, the macroscopicity measure $\bar{h}$ indicates the quantum character of the macroscopic system. Our results show that when the quantum nature of the potassium ion is evanesced, according to lower values of $\bar{h}$, the double-slit interference pattern becomes more classic. In other words, the values from $\bar{h} = 0.1$ to $\bar{h} = 0.01$ are more quantum values which makes the interference pattern possible, while the values lower than $\bar{h} = 0.01$ are classical values that show the interference pattern cannot be formed.

Molecular dynamics simulation

Our MD simulations [8, 9] are based on a model of the KcsA channel (Protein Data Bank, 1K4C.pdb), embedded in a palmitoyloleoyl phosphatidylcholine (POPC) lipid bilayer. The system was built from a cubic box of a 7.8 nm side that comprises KcsA (four subunits of 97 amino acids, 5292 atoms), water molecules (TIP3P model, 42296 atoms), 3K and 2K in the pore and 12 CL in the bulk (the entire system is electrically neutral). The AMBER 03 force field parameters [10] and GROMACS 4.5.3 software [11] was employed to perform the simulations with the time step of 1 fs. The protein was equilibrated during 10 ns in (N, V, T) then (N, P, T) ensembles. The temperature was kept at 300 K by Nose-Hoover coupling algorithm and the pressure was kept at 1 bar by Parrinello-Rahman coupling algorithm. The system is oriented along the z-axis. A cutoff was used for long-range interactions, namely: 1 nm for the van der Waals interaction and 1 nm for electrostatic interactions. Using the Particle-Mesh Ewald (PME) method, the electrostatic interactions are calculated. We have used the following abbreviations for amino acid identification: GLY1=GLY79, TYR=TYR78, GLY2=GLY77, VAL=VAL76, THR2=THR75, THR1=THR75. Each carbonyl group is a C=O compound in which the vibrations of C and O atoms are investigated separately. Taking typical values for membrane potentials in neurons, -70mV and +30mV, for resting and firing states are considered [12, 13]. To be more general, we also considered other membrane potentials -100 mV and +100 mV [14] to obtain other velocities as well [8, 9].

[1] Naeij, H. & Shafiee, A. Double-slit interference pattern for a macroscopic quantum system, *Found. Phys.* 10.1007/s10701-016-0034-7 (2016).
[2] Takagi S. Macroscopic Quantum Tunneling, Cambridge University Press, New York (2005).
[3] Merzbacher, E. Quantum Mechanics, Wiely, New York (1970).
[4] Holland, P. R. The Quantum Theory of Motion, Cambridge University Press, New York (1993).
[5] Zecca, A. Gaussian wave packets passing through two slits: contribution of confinement and tunneling to the diffraction pattern, *Adv. Studies Theor. Phys.* **7**, 287 (2013).
[6] Zecca, A. Two-slit diffraction pattern for Gaussian wave packets, *Int. J. Theor. Phys.* **38**, 911 (1999).
[7] Zecca, A. & Cavalleri, G. Gaussian wave packets passing through a slit: a comparison between the predictions of the Schrodinger QM and of stochastic electrodynamics with spin, *Nuovo Cimento* **112.B**, 1 (1997).
[8] Salari, V., Moradi, N., Fazileh, F. & Shahbazi, F. Quantum decoherence time scales for ionic superposition states in ion channels, *Phys. Rev. E* **91**, 032704 (1-6) (2015).
[9] Salari, V. *et al.* On the Classical Vibrational Coherence of Carbonyl Groups in the Selectivity Filter Backbone of the KcsA Ion Channel, *J. Integ. Neurosc.* **14(2)**, 195-206 (2015).
[10] Cornell, W. D. *et al.* A second generation force field for the simulation of proteins, nucleic acids, and organic molecules, *J. Am. Chem. Soc.* **117**, 5179-5197 (1995).

[11] Hess, B., Kutzner, C., van der Spoel, D. & Lindahl, E. GROMACS 4: algorithms for highly efficient, load-balanced, and scalable molecular simulation, *J. Chem. Theory Comput.* **4**, 435-447 (2008).
[12] Tegmark, M. Importance of quantum decoherence in brain processes, *Phys. Rev. E* **61**, 4194-4206 (2000).
[13] Waxman, S. G., Kocsis, J. D. & Stys, P. K. in The Axon (Oxford University Press, 1995).
[14] Kandel, E., Schwartz, J. & Jessell, T. Principles of Neural Science (McGraw-Hill, 2000).
